# Supplementary material for: Two missense mutations in KCNQ1 cause pituitary hormone deficiency and maternally inherited gingival fibromatosis
Source: Nat Commun. 2017 Nov 3;8:1289. doi: 10.1038/s41467-017-01429-z (PMC5668380; doi:10.1038/s41467-017-01429-z)
Supplement: Supplementary file 1 — Supplementary information [file 41467_2017_1429_MOESM1_ESM.pdf]

SUPPLEMENTARY NOTE 1: Clinical histories and phenotypic information.

All subjects described below have normal intelligence

1. The large pedigree from Northern Finland (Fig. 1a, pedigree I).

A. Patients born between 1991 and 2011

Subject 17

Subject 17 was born with normal weight (4000 g) and length (52 cm). As a child he had gingival fibromatosis, mild hypertelorism, thick ear lobules and cup-shaped ears, and broad nose. He displayed growth deceleration from +0.7 SDS (at birth) to -1.4 SDS at 3 years and -2.2 SDS at 5 years of age, when his bone age was 3.25 years. His IGF-1 levels were repeatedly low, yet his IGFB3 level was normal. His growth hormone response to insulin-induced hypoglycemia (peak GH concentration, 3.4 μg/l) and clonidine (maximum GH concentration, 2.4 μg/l) were both low. At the age of 5 years, GH deficiency diagnosis was set, treatment with growth hormone was started and continued until 18 years of age. The MRI scan of the brain revealed small hypophysis and thin stalk. He responded extremely well to GH and he achieved a final height of 188.9 cm. He had delayed puberty (at the age of 15 yr 11 mo, his Tanner stage was G2P1, his testosterone was <0.6 nM, inhibin B was 69 ng/l, and the LH response to GnRH stimulation were prepubertal: LH increased from <0.4 IU/L to 2.5 IU/L and FSH from 1.2 IU/L to 3.0 IU/L). To expedite puberty, he received testosterone treatment for 9 months. He was retested at the age of 17.5 years, and his clinical and hormonal findings were consistent with hypogonadotropic hypogonadism (testis volume 3.8 ml, serum T <0.6 nM, prepubertal LH response to GnRH: S-LH <0.4-2.6-2.9 IU/l and S-FSH 1.6-2.5-3.1 IU/l). He has not developed any signs of other pituitary hormone deficiencies.

Subject 18

Subject 18 was born after an uneventful pregnancy (birth weight 3470 g, birth length 47 cm corresponding to -1.8 SDS). She has a relatively mild gingival fibromatosis and a high-arched palate. During childhood, her height SDS was between -1.5 and -1.9. At the age of 12 years and 10 months, her bone age was 11 years. At the age of 13.4 years, her height was -2.3 SDS and the stage

of puberty was M3. She had low IGF-1 level (7.3 nM) and IGFBP3 level (2.6 mg/l). She had an absent GH response in the clonidine test (range of GH levels, 0.04-0.18 μg/l) and GH treatment was started. Her brain MRI scan was unremarkable. Her puberty started and progressed spontaneously, her menarche occurred at the age of 15 years and 3 months. Her adult height is 162.5 cm (-0.5 SDS). She has not developed other pituitary hormone deficiencies.

Subject 13

Her weight at birth was 2480 g and length was 52 cm. Unlike her father, she has not gingival fibromatosis or facial dysmorphia. At the age of one year, her height SDS was -0.3 and it decelerated to -2.7 at the age of 4 years, when she was referred to a pediatric endocrinologist. She had low GH response to insulin-induced hypoglycemia (the peak of serum growth hormone concentration, 1.8 μg/l) and to intravenous administration of L-dopa (the peak of serum growth hormone concentration, 0.9 μg/l). Cortisol response in the adrenocorticotropic hormone (ACTH) stimulation test was normal. At 4.2 years, her bone age was 3 years, and GH therapy was started at 4.5 years (at height SDS -2.7). Her brain MRI scan was unremarkable. Her puberty has started normally.

Subject 13b

She was born with normal birth weight (4340 g) and length (52 cm). Unlike her father, she does not have gingival fibromatosis or facial dysmorphia as a child. At the age of 1.8 years, her height was -1.5 SDS, and it declined further to -2.5 SDS at the age of 3.2 years. At that age, she had low GH response in insulin-arginine test (the peak of serum GH concentration, 2.78 μg/l), to L-Dopa stimulation (the peak of serum GH concentration, 0.77 μg/l), and delayed bone age. At the age of 3.7 years, when her height was -2.7 SDS, GH treatment was started. Her brain MRI scan was unremarkable. Concomitantly, she was diagnosed with central hypothyroidism, and thyroxine medication was commenced.

Subject 14

This subject has moderate gingival fibromatosis. His growth and pubertal development were normal, and his final height is 180 cm. He is healthy, and has not developed pituitary hormone deficiencies.

Subject 15

Subject 15’s birth weight was 2.9 kg and birth length was 47 cm, and he has gingival fibromatosis. At the age of 2 years, he was described to have cup-shaped ears, thick earlobes, and a high-arched palate. His height SDS was -1.2 SDS at the age of 3 years and then his growth decelerated to -1.9 SDS at 5 years, when his bone age was 3 years. He had a very low growth hormone response (0.68 μg/l) to hypoglycemia in the insulin tolerance test, a low GH response (4.29 μg/l) to clonidine, and low IGF-1(2 nmol/L). His brain MRI showed small hypophysis and thin stalk. He was diagnosed with GH deficiency, and he received GH treatment from the age of 6 years (-1.8 SDS) until the age of 16, and he achieved an adult height of 180 cm. Because of the delayed onset and slow progression of puberty, he has also received low-dose testosterone therapy. After withdrawal of GH treatment, he had weakness. Insulin tolerance test at 17 year of age revealed a unmeasurably low GH response, and GH replacement was continued at an adult dose for one year. As an adult, his facial features appear normal.

B. Patients born between 1963 and 1975

Subject 5

Subject 5 was born in the 1960’s at term with the birth weight of 3.5 kg and the birth length of 46 cm. As a child, she had gingival fibromatosis, delayed puberty (Tanner stage M1 at 13.5 years) and progressive growth deceleration: height standard deviation score (SDS) was -3.0 at 7 years; -3.6 SDS at 10; and -3.7 SDS at 13.5 years, when her bone age was delayed for 3 years). In 1977, at the age of 14.3 years, her growth hormone response to hypoglycemia was tested (peak GH, 9 μg/l) and watchful waiting was commenced. However, since her growth decelerated further (-4.5 SDS at 15

years of age with 3 years' delay in bone age) she received cadaveric hGH treatment for 13 months from Oct 1978 to Dec 1979. During the treatment, her puberty progressed, but menarche was delayed. Her final height is 147 cm (-3.7 SDS), which is > 2 SDS below her mid-parental target height (-1.6 SDS; mother's height 152 cm and father's height 171 cm). She was subsequently diagnosed with hypogonadotropic hypogonadism that was treated with sex hormone replacement therapy, which she had stopped at the age of 51. At the age of 52, she was clinically examined and her pituitary and adrenal functions were tested. Her BMI was 28.3 kg/m^2. Her estradiol levels, measured twice on consecutive days (0.05 nM and <0.04 nM), were low, as were her baseline FSH (8.2 IU/L) and LH (4.1 IU/L) levels for such low estradiol levels[1]. However, the results of the GnRH stimulation test suggested that some of her pituitary gonadotropin secretion capacity was preserved (peak FSH, 11.2 IU/L; peak LH, 13.3 IU/L). Her IGF-1 level was low, 5.2 nmol/L (cutoff of 16.7 nmol/l has 85% sensitivity and 68% specificity for the diagnosis of GHD[2]. Her GH secretion response to arginine (<0.05-0.30-0.48-0.21-0.10 μg/l) was low and the peak response (0.48 μg/l) was <1.4 μg/l, a cutoff for diagnosing GHD with 95% sensitivity and 62% specificity (a peak value <0.4 μg/l would have had 87% sensitivity and 91% specificity) for the diagnosis of adult GHD[2]. Her response to exogeneous GRF (<0.05-1.19-0.54-0.40-0.30 μg/l) was low and the peak value (1.19 μg/l) was < 2.7 μg/l (the lowest observed peak value in 178 healthy adults following GHRH stimulation)[3]. Her brain MRI scan was unremarkable. She is currently on thyroxine medication that was started in primary care on the basis of slightly elevated TSH level in the setting of normal free thyroxin. Her cortisol secretion was normal. She does not have children.

Subject 6

Subject 6 was born in the 1960's with normal body weight (3.69 kg) and length (51 cm). She had gingival fibromatosis, and as she displayed growth deceleration during childhood (-2 SDS at the age of 2 years; -2.5 SDS at 11 years; and -3.4 SDS at 12.4 years of age). Her GH response to hypoglycemia was tested in 1977, at the age of 11.6 years, with a peak GH response of 6.5 μg/l. She

subsequently received cadaveric hGH treatment from the age of 12.4 years approximately one year (from Jan 1978 to Mar 1979). Her adult height is 148 cm (-3.5 SDS), which is 1.9 SDS below the mid-parental target height. Her menarche occurred at 15 years of age. Later in life, she suffered from infertility, attempts to induce ovulation with clomiphene citrate were unsuccessful and she does not have children. She has used frequent progestin therapy for menstrual cycling. She uses regular thyroxine medication that was started in primary healthcare. She has rheumatoid arthritis that is treated with glucocorticoids and abatacept, and asthma that is treated with inhaled salmeterol and fluticasone. At the age of 50 years, her pituitary function was retested. At that time her BMI was 22.8 kg/m^2. On clinical examination she has mild gingival hyperplasia. Her circulating estradiol level measured twice on consecutive days from early morning samples was low (<0.04 nmol/l) and her FSH (7.0 IU/L) and LH (4.2 IU/L) levels were low for estradiol[1]. The results of the GnRH stimulation test suggested that some of her pituitary gonadotropin secretion capacity was preserved (peak FSH, 10.7 IU/L; peak LH, 18.3 IU/L). Her IGF-1 level was low, 4.9 nmol/L, as were her GH secretion responses to arginine (<0.05-0.37-0.83-0.28-0.14 μg/l) and GHRH (0.10-0.56-0.28-0.21-0.12 μg/L) (see discussion above for Subject 5 for interpretation of IGF-1 and stimulation test values). Her brain MRI scan was unremarkable

Subject 7

The smaller (birth weight, 1.6 kg; birth length 42.5 cm) of the identical twin boys (Subjects 7 and 8) was born in breech position after a full-term pregnancy in the 1960's. As a child, he had prominent gingival fibromatosis, broad nostrils, and a progressive growth failure: at the age of 1.5 years, his height was at -2.0 SD; at 7.7 years it was -4.8 SDS and at the age of 8.5 years it was -5.0 SDS. His bone age was four years delayed (4.5 years at the age of 8.5 year). In 1976, growth hormone deficiency was diagnosed based on low GH response in insulin-arginine test (peak GH, 3.0 μg/L), and GH treatment was started. He responded well to the treatment (his adult height is 176 cm). He had a delayed TSH response in the TRH stimulation test, and, at the age of 11 years thyroxine was started. Fluoxymesterone, an androgen used to induce puberty in Finland in the 1980s, was started

at 12 years. One year later, he complained about extreme fatigue and was subsequently treated with regular hydrocortisone medication, which completely resolved his symptoms and the diagnosis of adrenal insufficiency was set. At the age of 45 years, he was on daily thyroxine, hydrocortisone, and growth hormone medications and used testosterone undecanoate at 3-month intervals. He had slight cubitus valgus, an extension deficit in the elbows, and no facial dysmorphism. At that time, he had seven naturally conceived children. Clinical examination revealed small testicular size (4 ml), low inhibin B (31 ng/l), and low serum follicle-stimulating hormone (FSH) (2.6 IU/l) and low-normal luteinizing hormone (LH) level (2.2 IU/l), findings consistent with partial hypogonadotropic hypogonadism. His brain MRI scan revealed a small pituitary gland.

Subject 8

He was born at term (birth weight 2.6 kg; birth length 46 cm), and has gingival fibromatosis, and similar facial features as his identical twin brother (Subject #7). His puberty was induced with testosterone. At the age of 15 years 9 months, he had an absent growth hormone response to intravenous clonidine stimulation, diagnosed with growth hormone deficiency, and growth hormone treatment was started for 3 months at the age of 15 years and 11 months when his height was -2.6 SDS. Seventeen months later, his growth hormone secretion capacity was re-tested with an intravenous growth hormone-releasing factor, to which he responded normally (peak stimulated GH concentration of 17 μg/l) and he received another growth hormone course for 1.5 years. He is nine centimeters shorter, 167 cm, than his identical twin brother who had had much more profound growth deflection and who had received GH Rx for many years. At 21 years of age, he was diagnosed with central hypothyroidism. At 45 years, he is receiving testosterone and thyroxine replacement therapy, and he does not have children.

Subject 9

Subject 9’s weight at birth was 2.9 kg and her length was 47 cm. As a child she was noted to have gingival fibromatosis, broad nose, and high-arched palate. She grew normally until the age of 6

years (at the age of 4, her growth was -0.7 SDS), when her growth almost completely ceased[4] for almost a year. Then her growth recovered gradually, and at 9 years her height was -2.7 SDS. She was diagnosed with GH deficiency on the basis of a low GH response (maximum 2.4 μg/l) to intravenous clonidine administration. She displayed a GH response (peak stimulated growth hormone level, > 20 μg/l) to growth hormone-releasing factor[4]. She received GH replacement therapy from 9 to 15 years of age to which she responded well, and she is clearly taller than her sisters (Subjects 5 and 6) with a final height of 154 cm (-2.3 SDS). She had delayed puberty, and her puberty was induced by estradiol valerate. From the age of 16, she has been receiving estradiol-progestin combination therapy.

2. The Finnish trio; subjects 20-22 (Fig. 1a, pedigree II)

Subject 20 was born with normal birth weight (3500 g) and length (49.5 cm). She was adopted at the age of 4 months, and information on her parents is not available. She exhibited a severe growth failure from -3 SDS at 2 years of age to -4.1 SDS at 8 years and to -5.2 SDS (137 cm) at 17 years with delayed bone age (14 years). Serum IGF-1 level was low, and insulin-arginine-ACTH stimulation test results were consistent with classic GH deficiency (GH response 0.4-0.4-0.3-0.3 μg/l); her cortisol response was normal (from 681 to 993 nM). Her puberty was stalled (Tanner stage M3). She had one café au lait spot, small hands and feet, and numerous nevi, but no gingival fibromatosis. Her karyotype is 46, XX. Ultrasound revealed an infantile uterus (length, 2 cm) and small ovaries. Brain MRI was normal. Human GH treatment was started at the age of 17.3 years. Three months later, the length of uterus had increased to 5.3 cm; 9 months later, her Tanner stage was M5P5 and she had menarche. At 18 years, she had a delayed TSH response to TRH stimulation in two different occasions. At 19.5 years, GH therapy was stopped and a few months thereafter she developed secondary amenorrhea and was lost to follow-up for many years. Her adult height is 146.1 cm (-3.8 SDS). At the age of 30, she had an appointment with an adult endocrinologist and was diagnosed with an adrenal insufficiency (baseline cortisol, 110 nM; stimulated cortisol 195

nM), central hypothyroidism, and GH deficiency (serum IGF-1 was 5 nM; arginine-GHRH stimulation test results revealed a low baseline (< 0.05 mg/l) and very low maximal GH level of (0.6 mg/l); a cutoff of 4.1 mg/l for the peak stimulated GH level would detect GHD with 95% sensitivity and 91% specificity[2]. Subsequently, hydrocortisone, thyroxine, and GH treatments were started. She has retinal pigmentation (**Supplementary Fig. 3**).

At the age of 27 years, she became pregnant (dichorial and diamnionic) after ovulation induction, and gave birth to twin boys at 33 weeks of gestation. Both boys had gingival fibromatosis. The smaller of the newborn boys (**subject 21**) weighed 1290 g, his length was 38 cm, and he had unilateral cryptorchidism. At the age of 2.7 years, he displayed short stature (-3.0 SDS), a subnormal GH response in arginine stimulation test (GH peak, 2.6 mg/l), and, subsequently, GH treatment was started. He has a long philtrum, a broad nose with anteverted nostrils, and thick ear lobules (**Fig. 1b**). At 8 years, his prolactin level was 347 mU/l (ref. range 50-300 mu/L). At 10, his GH response to arginine stimulation was again low (1.47-0.58-0.49-3.29 mg/l), yet he exhibited a robust response to exogeneous GRF (stimulated max. 14.2 mg/l), suggesting some pituitary reserves for GH. His testosterone was 1.6 nM, and he had normal results in ACTH stimulation test. He has not shown any signs of additional pituitary hormone deficiencies or pigmentation defects. His puberty has started normally. Brain MRI revealed small hypophysis.

His twin brother (**subject 22**) weighed 2400 g at birth, and had more pronounced hypertelorism than his brother, slightly downward slanting palpebral fissures, a long philtrum, a broad nose with anteverted nostrils, thick ear lobules, and gingival fibromatosis. During childhood, his growth has been steady and proceeded at -1.5 SDS. At the age of 9 years, his bone age was 7.8 years. Due to his family history and gingival phenotype, his growth hormone secretion capacity was tested at the age of 4 years, when he displayed a low response to i.v. arginine (3.2-0.53-0.6-2 mg/l). He was retested at the age of 10 years, when he again displayed profound GH deficiency in the arginine stimulation test (<0.05-1.0-0.66-0.11 mg/l), but had some pituitary GH reserves (peak GH

level following exogeneous GRF, 5.67 μg/l). Although he has evidence of biochemical GH deficiency, he grows normally and therefore GH therapy has not yet been started.

3. Family originating from Argentina: subject 25 (Fig. 1a, pedigree III)

The index patient in this family (subject 25) had gingival fibromatosis (operated 3 times in childhood) and has a short adult stature (149.4 cm). Her parents did not have gingival fibromatosis or pituitary hormone deficiencies. She has not been treated with GH. She reported growth arrest at the age of 11-12 years, when she also had her menarche. At the age of 18 years, she had surgery for maxillary malformation and concomitantly lost weight with transient secondary amenorrhea. Her menstrual cycles recovered 1 year after the surgery. Her brain MRI scan was normal. She has refused to test her anterior pituitary function. She has two daughters, both with gingival fibromatosis. The older girl was born by caesarean section at 41 weeks of gestation (BW 3350 g, BH 47 cm). At the age of 5 years, she was diagnosed with classical GH deficiency. MRI examination revealed a small pituitary gland and an asymmetric posterior part of the gyrus rectus. She has similar facial features to subjects 21 and 22, including large nostrils and thick earlobes. Her younger sister (by 2.5 years) was born small for gestational age, had gingival fibromatosis (operated twice), and so far has displayed a normal growth rate. DNA analyses of the two girls are not available.

SUPPLEMENTARY FIGURES

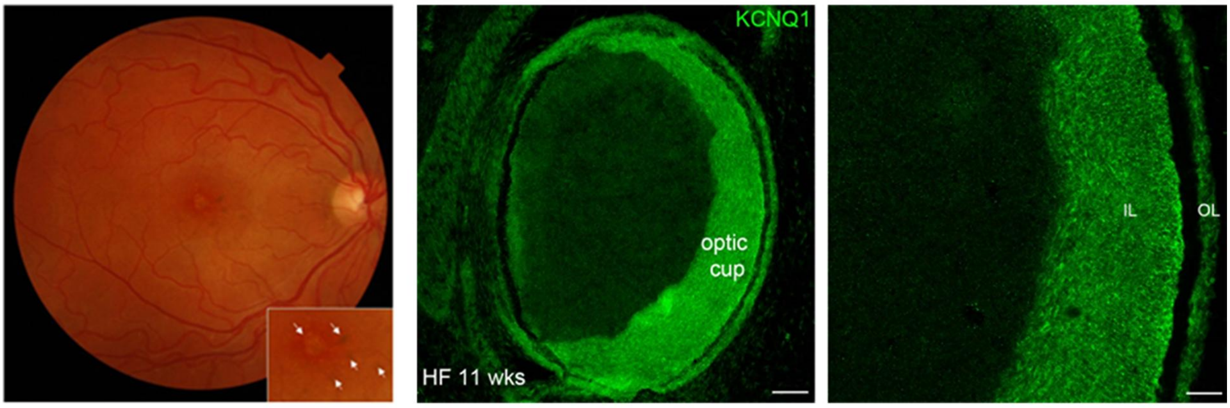

Supplementary Figure 1 Retinal pigmentation and KCNQ1 immunostaining in the optic cup *Left panel* shows the pigmentary changes in the macula, present in proband #20 carrying the p.Pro369Leu mutation in *KCNQ1*; close-up of the central macula with arrows pointing to the small pigment clumps and depigmented areas. *Middle* and *right panels* show a representative coronal section of a human fetus at 11 weeks post-amenorrhea, immunolabeled for KCNQ1. Strong KCNQ1 immunofluorescence is detectable in the optic cup (OC) (*middle panel*), formed by the inner (IL) and the outer layers (OL) shown in the right panel. Scale bar in middle panel indicates 100 mm, scale bar in left panel indicates 40 mm.

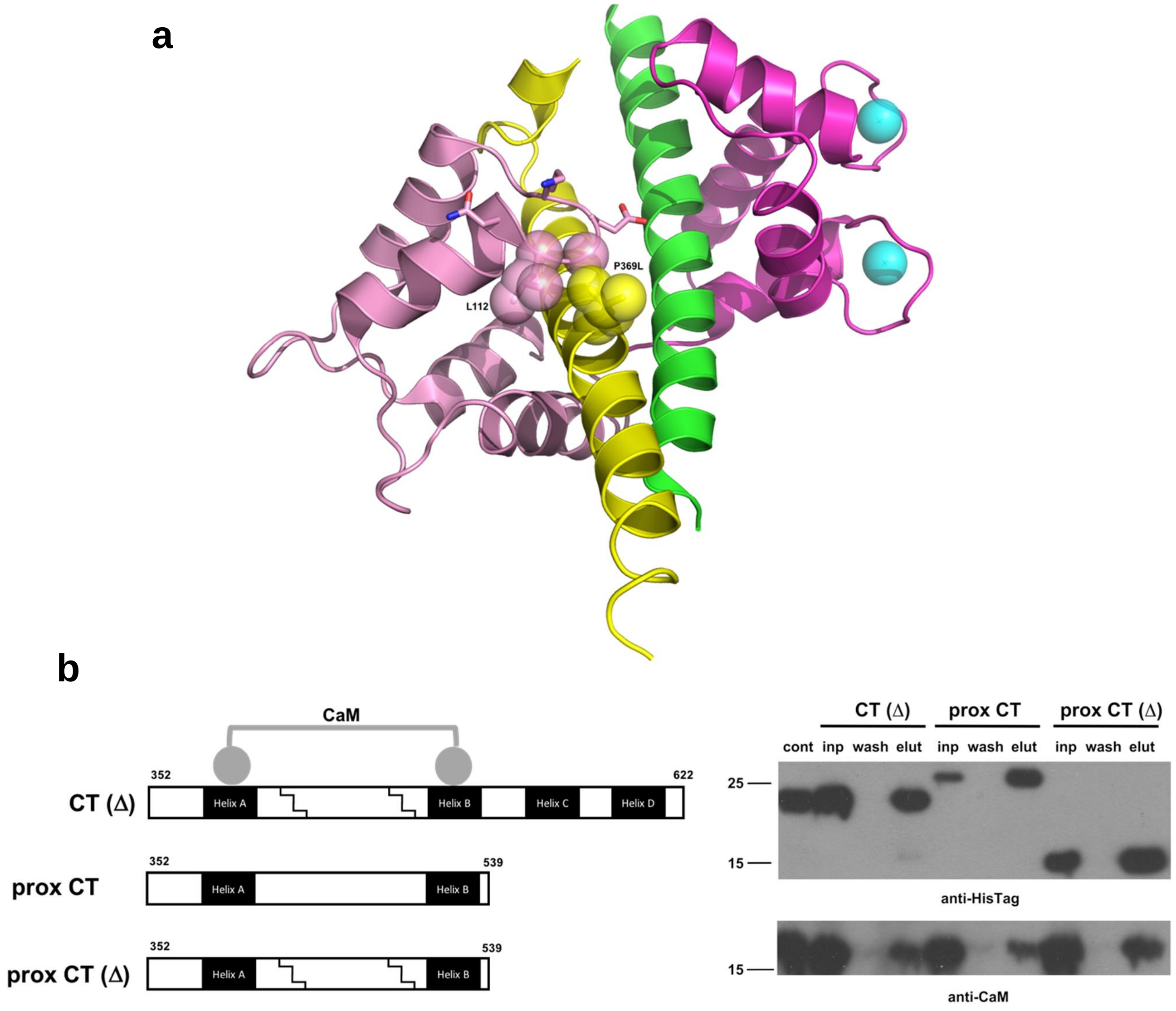

Supplementary Figure 2 The Pro369Leu mutation does not compromise the structural integrity of the proximal C-terminal (CT)/CaM complex

(a) In this molecular model, the anti-parallel coiled-coil of the KCNQ1 proximal C-terminus (helix A colored in yellow; helix B colored in green) is embraced by CaM (N-lobe colored in magenta; C-lobe colored in pink). Bound to the CaM N-lobe are two Ca^{2+} ions, shown as cyan spheres. Pro369Leu is depicted in CPK representation along with residues Leu112 and Gly113 from the CaM C-lobe loop likely to interact with the mutated Leu. No steric occlusion is observed due to introduction of the mutant, CaM interaction with the proximal CT should not be compromised. **(b)** Biochemical characterization of the Pro369Leu mutant using a pull-down assay of the intracellular CT to test

binding of CaM. In earlier studies, we established a recombinant bacterial co-expression system for the Kv7.1 CT in a complex with CaM[5,6]. In this system, we can purify various forms of CT and CaM as a stable complex. We used this system to assess whether Pro369Leu disrupted CaM association. The left panel shows the constructs tested with the Pro369Leu point mutation introduced. All constructs have an N-terminal HisTag which was used for pulling down the CT. CT (D) is HisTag KCNQ1 352-622 H620A with a deletion of Δ406-504, indicated by zig-zags. This region of the protein is a long intervening loop between helices A and B that is not conserved and is not required for function[5]. CT (D) forms a hetero-octameric assembly of 4 Kv7.1 CT and 4 CaM molecules[6]. The proximal CT is HisTag KCNQ1 352-539 and contains the intervening loop without the distal CT, but contains the CaM binding site (helices A and B), forming a hetero-dimer (CT/CaM). The proximal CT (D) is HisTag KCNQ1 352-539 Δ406-504 and represents the minimal proximal CT that associates with CaM. We tested these three configurations to comprehensively address the potential structural context of CaM binding perturbation, i.e. with or without an intervening loop between helices A and B and whether as a dimer with the proximal CT or as an oligomeric assembly. The right panel displays the results of the pull-down assay for all three constructs. Bacterial lysate was prepared (inp), incubated with metal-chelate beads. Beads were stringently washed with buffer (wash), and eluted with imidazole (elut). Samples were run on SDS-PAGE. Immunoblots were performed on pull-downs of the three KCNQ1 mutant Pro369Leu CT constructs and WT CT (D) that serves as a marker (cont). Molecular weight standards (kDa) are denoted. In all cases, CaM (lower panel) was found bound to the CT protein, demonstrating stable association. The assay was repeated twice. We conclude that Pro369Leu does not affect CaM binding in the varying quaternary structure contexts tested. The blot has been cropped for clarity. The full-length blot is presented in Supplementary Figure 3.

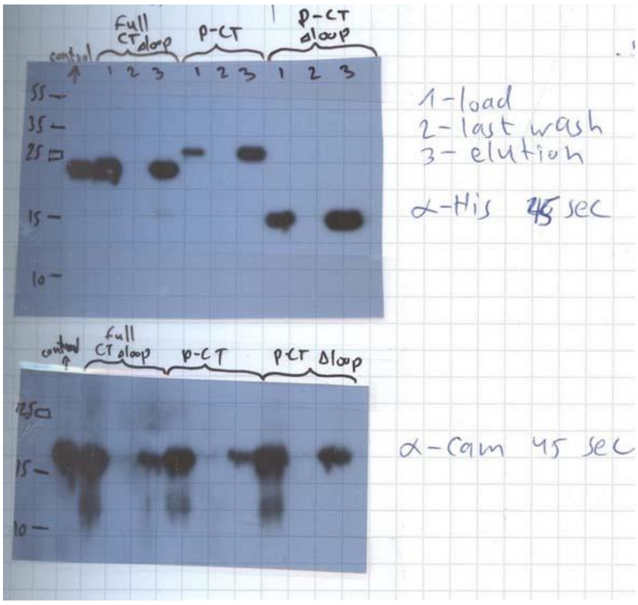

Supplementary Figure 3 The full-length blots for the pull-down assay of the intracellular CT to test binding of CaM

Bacterial lysate was prepared (1-load), incubated with metal-chelate beads. Beads were stringently washed with buffer (2-wash), and eluted with imidazole (3-elution). Samples were run on SDS-PAGE. Immunoblots were performed on pull-downs of the three KCNQ1 mutant Pro369Leu CT constructs and WT CT (Δ) that serves as a marker (control). Molecular weight standards (kDa) are denoted.

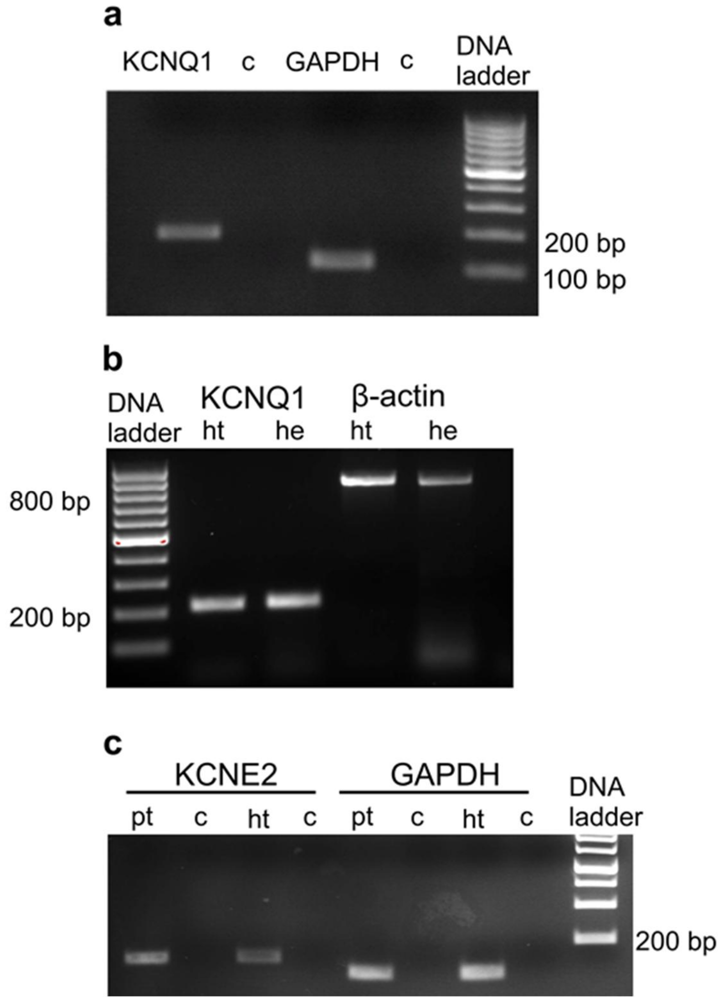

Supplementary Figure 4 RT-PCR analysis of *KCNQ1* and *KCNE2* expression in human pituitary gland (a) and (b) hypothalamic cDNA

The PCR products were visualized on a 1.5% agarose gel. (a) A 183-bp fragment of transcript encoding KCNQ1 was amplified from human pituitary gland cDNA. (QUICK-Clone cDNA, Clontech, 1.5µl / reaction) Human *GAPDH* was used as a reference gene. c: negative control without DNA template. (b) A 213-bp fragment of transcript encoding KCNQ1 was amplified from human hypothalamic cDNA, or, as a positive control, from a cardiac cDNA library. Human β-actin was used as a reference gene. ht, hypothalamus; he, heart. (c) A 172-bp fragment of transcript encoding KCNE2 was amplified from human pituitary gland cDNA and hypothalamic cDNA. Human GAPDH was used as a reference gene. pt, pituitary gland; ht, hypothalamus; c, negative control without DNA template.

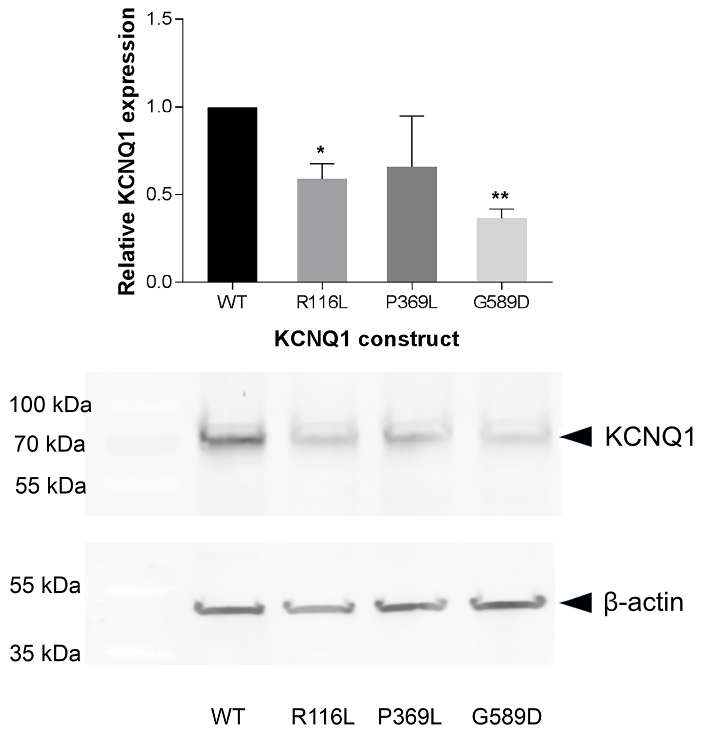

Supplementary Figure 5 Quantification of WT and mutant KCNQ1 expression in AtT-20 cells
The relative expression of the different mutant KCNQ1 constructs in relation to WT KCNQ1 was quantitated from three separate experiments by using Image Studio Lite software. β-actin served as a protein loading control and was imaged from the same blots as KCNQ1. The mean (±SD) of three independent experiments are shown. *P<0.05 **, P<0.005 (t-test against WT KCNQ1, which was set to 1.00). A representative western blot used in the quantification is also presented. The western blots have been cropped for clarity. The full-length blots are presented in Supplementary Figure 6.

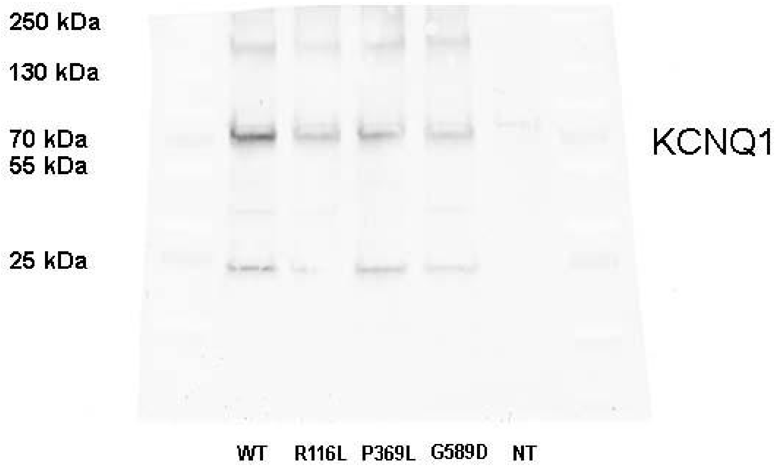

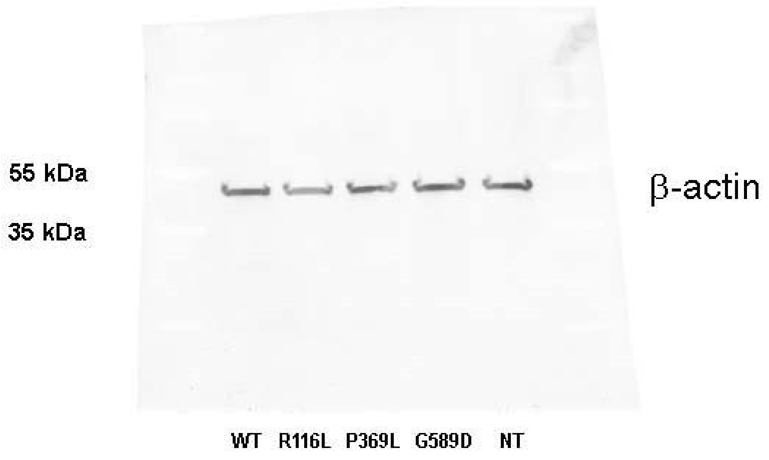

Supplementary Figure 6 The full-length blots for the Western blotting of KCNQ1 from AtT-20 cells transfected with KCNQ1 and KCNE2

β-actin served as a protein loading control and was imaged from the same blots as KCNQ1. Molecular weight standards (kDa) are denoted. NT, not transfected.

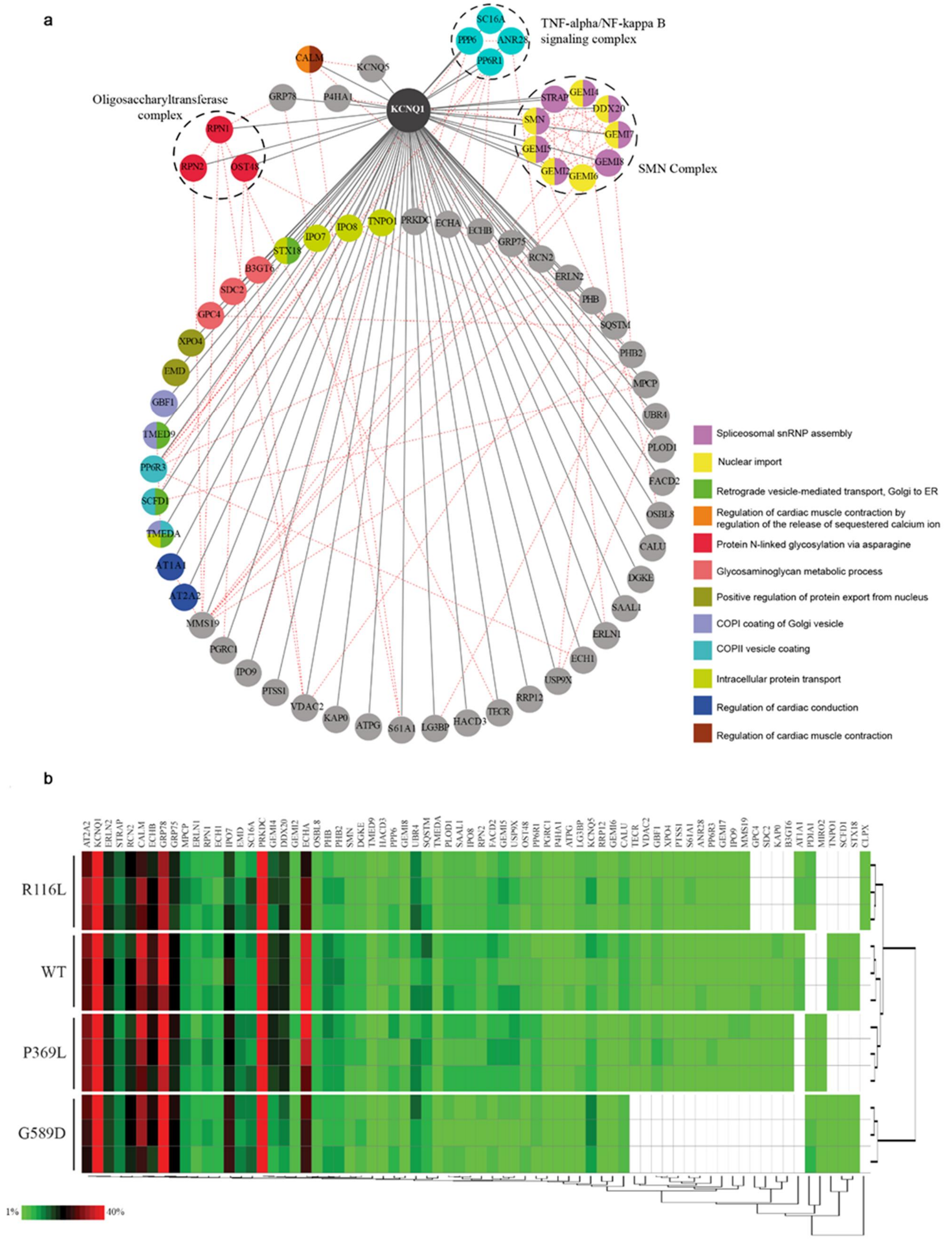

Supplementary Figure 7 Interactome analysis reveals known and novel interactions for KCNQ1

(a) Affinity purification mass spectrometry analysis of KCNQ1 identified 68 high-confidence protein-protein interactions (solid grey lines). Enriched Biological processes in which the KCNQ1 interactors are involved are illustrated with node fill color. The proteins involved in the same protein complex (CORUM database) are grouped (dashed circles). Known prey-prey interactions are shown with dashed red edges.

(b) Hierarchical clustering of quantitative AP-MS analysis of KCNQ1 and the patient mutations. The heatmap color gradient (1%-40%) illustrates the corresponding interactor abundance relative to the KCNQ1 bait protein.

Supplementary Table 1 The primers used in *KCNQ1* Sanger sequencing and in the RT-PCR-amplification of *KCNQ1* and *KCNE2*

**Primers used for the Sanger sequencing of the *KCNQ1* (5' → 3')**		
KCNQ1ex1ATG_F	GTGGCTGCCCGCACT	
KCNQ1ex1ATG_R	ACCCCAGCGCTTCCTCT	
KCNQ1ex1AF	CCCTCCTCGTTATGGCCG	
KCNQ1ex1AR	TGCTGTAGATGGAGACGCG	
KCNQ1ex1BF	CCCTTCTCGCTGGAGCTG	
KCNQ1ex1BR	ACTTCCTTCCCTCCTCTGCT	
KCNQ1ex2F	AATGGATGACTGGGTTTTCG	
KCNQ1ex2R	TATCAGGGCAGGACCAATGT	
KCNQ1ex3F	AGCATGGCTGGGTTCAAA	
KCNQ1ex3R	ACTCCATCTGGTAGGGGTCA	
KCNQ1ex4F	ACGAGAGCAGGGTGTATGCT	
KCNQ1ex4R	GTGGATGGGGCGTGAGAC	
KCNQ1ex5F	AGGGACACCCATGCCATC	
KCNQ1ex5R	CGCATCTCAAGCTGTCCTAGT	
KCNQ1ex6F	GCCACTTACCGGAGTTGTGA	
KCNQ1ex6R	CAAGACACCAGTGCCCAGAT	
KCNQ1ex7F	TCATCAGAGTGGTGGGTTTG	
KCNQ1ex7R	CGTAAGTGGGTCTGCTCACA	
KCNQ1ex8F	ATACCTGGCCTTCCCACAAC	
KCNQ1ex8R	CCAATGATGGTTCTGACAGG	
KCNQ1ex9F	GGGGAGCTGTAGCTTCCATA	
KCNQ1ex9R	AGCCAAATGCATGGTGAGAT	
KCNQ1ex10F	CTGCCCTGTCTCTGTGTGAA	
KCNQ1ex10R	GGAGAAACTCACTGGCTTGC	
KCNQ1ex11F	ACTGATTGTCAGGGCTGGAG	
KCNQ1ex11R	TGGGCACTAGGCGAGTAGAT	
KCNQ1ex12F	TCTGGAAGGATCCAGTCTGC	
KCNQ1ex12R	CTCCACTATGGGCAGGAGAG	
KCNQ1ex13F	AACCAGGCTTATGCCATCAC	
KCNQ1ex13R	GGTGGTTGAGAGGCAAGAAC	
KCNQ1ex14F	CCCAGAGTGGGTGGACAGT	
KCNQ1ex14R	ATGGCCCATTCTGACATCAT	
KCNQ1ex15F	TGTCCTAGTAGGTTTAGGCATTTTG	
KCNQ1ex15R	CTTCACGTTCACACGCAGAC	
KCNQ1ex16F	GTTGGCACCTTCCCTTCTCT	
KCNQ1ex16R	ACTCTTGGCCTCCCCTCTC	
**Primers used for the RT-PCR (5' → 3')**		expected size (bp)
HuKCNQ1_F	GCCCCAAACCCAAGAAGTCT	213
HuKCNQ1_R	TGAAATGGGGCATGCTCACT	
HuKCNQ1_F2	AGCTACGCAGATGCGCTGT	186
HuKCNQ1_R2	CTGCTTCTGCTGCACCTTC	
HuGAPDH_F	CTCTCTGCTCCTCCTGTTCG	111
HuGAPDH_R	ACCAAATCCGTTGACTCCGA	
HuKCNE2_F	GAATTTCATCCTGCCCACAC	172
HuKCNE2_R	TCAGCATCAACTTTGGCTTG	

Supplementary references

1. Overlie I, Mørkrid L, Andersson AM, Skakkebaek NE, Moen MH, Holte A. Inhibin A and B as markers of menopause: a five-year prospective longitudinal study of hormonal changes during the menopausal transition. Acta Obstet Gynecol Scand. 2005;84:281-5
2. Biller BM, Samuels MH, Zagar A, Cook DM, Arafah BM, Bonert V, Stavrou S, Kleinberg DL, Chipman JJ, Hartman ML. Sensitivity and specificity of six tests for the diagnosis of adult GH deficiency. J Clin Endocrinol Metab. 2002;87:2067-79
3. Aimaretti G, Baffoni C, DiVito L, Bellone S, Grottoli S, Maccario M, Arvat E, Camanni F, Ghigo E. Comparisons among old and new provocative tests of GH secretion in 178 normal adults. Eur J Endocrinol. 2000;142:347-52
4. Oikarinen, K., Salo, T., Käär, M.L., Lahtela, P. & Altonen, M. Br. J. Oral Maxillofac. Surg. 28, 335–339 (1990).
5. Shamgar, L. *et al. Circ. Res*. **98,** 1055-1063 (2006).
6. Wiener, R. et al. J. Biol. Chem. 283, 5815-5830 (2008).
